# Ectopic Expression of *GsSRK* in *Medicago sativa* Reveals Its Involvement in Plant Architecture and Salt Stress Responses

**DOI:** 10.3389/fpls.2018.00226

**Published:** 2018-02-22

**Authors:** Mingzhe Sun, Xue Qian, Chao Chen, Shufei Cheng, Bowei Jia, Yanming Zhu, Xiaoli Sun

**Affiliations:** ^1^Plant Bioengineering Laboratory, College of Life Science, Northeast Agricultural University, Harbin, China; ^2^Crop Stress Molecular Biology Laboratory, Agronomy College, Heilongjiang Bayi Agricultural University, Daqing, China

**Keywords:** wild soybean, alfalfa, receptor-like kinase, S-locus LecRLKs, salt stress, plant architecture

## Abstract

Receptor-like kinases (RLK) play fundamental roles in plant growth and stress responses. Compared with other RLKs, little information is provided concerning the S-locus LecRLK subfamily, which is characterized by an extracellular G-type lectin domain and an S-locus-glycop domain. Until now, the function of the G-type lectin domain is still unknown. In a previous research, we identified a *Glycine soja* S-locus LecRLK gene *GsSRK*, which conferred increased salt stress tolerance in transgenic *Arabidopsis*. In this study, to investigate the role of the G-type lectin domain and to breed transgenic alfalfa with superior salt stress tolerance, we transformed the full-length *GsSRK* (*GsSRK-f*) and a truncated version of *GsSRK* (*GsSRK-t*) deleting the G-type lectin domain into alfalfa. Our results showed that overexpression of *GsSRK-t*, but not *GsSRK-f*, resulted in changes of plant architecture, as evidenced by more branches but shorter shoots of *GsSRK-t* transgenic alfalfa, indicating a potential role of the extracellular G-type lectin domain in regulating plant architecture. Furthermore, we also found that transgenic alfalfa overexpressing either *GsSRK-f* or *GsSRK-t* showed increased salt stress tolerance, and *GsSRK-t* transgenic alfalfa displayed better growth (more branches and higher fresh weight) than *GsSRK-f* lines under salt stress. In addition, our results suggested that both *GsSRK-f* and *GsSRK-t* were involved in ion homeostasis, ROS scavenging, and osmotic regulation. Under salt stress, the Na^+^ content in the transgenic lines was significantly lower, while the K^+^ content was slightly higher than that in WT. Moreover, the transgenic lines displayed reduced ion leakage and MDA content, but increased SOD activity and proline content than WT. Notably, no obvious difference in these physiological indices was observed between *GsSRK-f* and *GsSRK-t* transgenic lines, implying that deletion of the *GsSRK* G-type lectin domain does not affect its physiological function in salt stress responses. In conclusion, results in this research reveal the dual role of *GsSRK* in regulating both plant architecture and salt stress responses.

## Introduction

Alfalfa (*Medicago sativa*) is an important perennial leguminous forage, and is widely cultivated in the world (Yu, 2017). However, alfalfa is a moderately salt-tolerant crop; its production is severely limited by salt stress (Yu et al., 2016a; Arshad et al., 2017; Liu and Yu, 2017). Unfortunately, approximately 954 million hectares of the global land are sodic soil, which largely restricts alfalfa cultivation and forage production (An et al., 2016). Therefore, breeding of salt-tolerant alfalfa varieties is a dire need to expand its planting area.

Wild soybean (*Glycine soja*) belongs to the same family *Leguminosae* together with *Medicago sativa*. What is different is that wild soybean is highly tolerant to salt stress (Joshi et al., 2013; Zhang et al., 2016a). In previous researches, we screened out a salt-alkaline tolerant wild soybean variety G07256, and identified several salt stress responsive genes (Zhu et al., 2011; Wang et al., 2012; Yang et al., 2012; Sun et al., 2014, 2016a,b; Yu et al., 2016b). For instance, *GsZFP1* (Tang et al., 2013), *GsTIFY10a* (Zhu et al., 2014), *GsACA1* (Sun et al., 2016a) overexpression in alfalfa increased the salt tolerance of transgenic plants. Hence, wild soybean is an ideal candidate for identifying salt resistant genes, which will facilitate the molecular breeding of transgenic alfalfa with superior salt tolerance.

The receptor-like kinase (RLK) family is a major protein kinase family, with 610 members in *Arabidopsis*, 1132 in rice, and 1418 in soybean (Shiu et al., 2004; Gish and Clark, 2011; Liu et al., 2015). A typical RLK protein contains a C-terminal intracellular kinase catalytic domain, a transmembrane domain and an N-terminal extracellular domain (Shiu and Bleecker, 2003; Ye et al., 2017). According to the architecture of their extracellular domains, RLKs are divided into 44 different subfamilies (Shiu and Bleecker, 2003). Among them, the S-locus LecRLK subfamily is characterized by a signal peptide, a G-type lectin domain, an S-locus-glycop domain, and a PAN-AP domain within the extracellular domain. The S-locus-glycop domain is reported to be linked to self-incompatibility (Zhang et al., 2011). Furthermore, the S-locus-glycop and PAN-AP domains are responsible for dimerization of the S-locus LecRLKs (Naithani et al., 2007). However, the biological function of the G-type lectin domain is yet to know.

Current researches have demonstrated the important regulatory role of RLKs in plant stress responses (Marshall et al., 2012; Vaid et al., 2013; Ye et al., 2017). For instance, *OsSIK1*, a rice LRR-RLK gene, positively regulates plant tolerance to salt stress (Ouyang et al., 2010). Overexpression of *GsCBRLK*, a Ca^2+^/CAM binding RLK from wild soybean, could increase salt tolerance of transgenic *Arabidopsis* and alfalfa (Yang et al., 2010; Bai et al., 2013). In addition, *GsRLCK*, a *Glycine soja* cytoplasmic RLK gene, also conferred increased salt tolerance (Sun et al., 2013a). However, compared with other RLKs, little is known regarding to the function and molecular basis of the S-locus LecRLKs in plant stress responses.

Our previous research identified a *Glycine soja* S-locus LecRLK, *GsSRK*, which conferred enhanced salt stress tolerance in transgenic *Arabidopsis* (Sun et al., 2013b). Hence, to investigate the role of the G-type lectin domain in stress responses and to breed salt-tolerant transgenic alfalfa, we transformed the full-length *GsSRK* (*GsSRK-f*) and a truncated version (*GsSRK-t*) deleting the G-type lectin domain into alfalfa, and evaluated the salt stress tolerance of transgenic alfalfa. Our results showed that overexpression of *GsSRK-t*, but not *GsSRK-f*, resulted in morphological changes, indicating a potential role of the extracellular G-type lectin domain in regulating plant architecture. Furthermore, both *GsSRK-f* and *GsSRK-t* overexpression increased salt stress tolerance by regulating ion homeostasis, ROS scavenging, and osmotic stress. Notably, even though no obvious difference in the physiological indices was observed between *GsSRK-f* and *GsSRK-t* transgenic lines, *GsSRK-t* overexpression conferred better growth of transgenic alfalfa under salt stress than *GsSRK-f*, suggesting a potential use of *GsSRK-t* in breeding salt-tolerant alfalfa.

## Materials and Methods

### Multiple Alignment of GsSRK and Its Homologous Proteins

The full length amino acid sequences of GsSRK and its homologous proteins were used for multiple alignment by using Clustal software (Chenna et al., 2003). The position of the conserved domains was marked according to GsSRK protein sequence. The accession numbers for GsSRK homologous proteins were Medtr8g061110, LOC_Os07g08860 (OsSIK2), LOC_Os01g47900 (OsLSK1), At1g11350 (AtCBRLK1/RKS2), and At1g65800 (AtARK2).

### Construction of the *GsSRK* Overexpression Vectors

The full-length coding region of *GsSRK* (2505 bp) was amplified according to the homologous genes (Accession No.: Glyma.06G255900) in cultivated soybean, by using the gene specific primers (5′-ATGGCATTCACTTCTGCCCT-3′ and 5′-TACATAAGGAGAAATGGGGGG-3′). The truncated *GsSRK* without the N-terminal extracellular signal peptide and G-type lectin domain (*GsSRK-t*, without 471 bp in the 5′ end) was amplified with the following primers (5′-ATGAATCTAGGATATAATTCCG-3′ and 5′-TCATCTTGCTTCCACCATT-3′). The PCR product was purified and inserted into the *Sma*I-digested pBEOM vector (Sun et al., 2016a) under the control of the CaMV35S promoter and the E12 enhancer. The resulting constructs pBEOM-GsSRK-f and pBEOM-GsSRK-t were subjected to sequencing.

### Transformation and Identification of the *GsSRK* Overexpression Transgenic Alfalfa

The wild type (WT) *Medicago sativa* L. (ZhaoDong cultivar) was used for generating *GsSRK* overexpression lines. Cotyledonary nodes of alfalfa were cut from the 7-day-old aseptic seedlings, and were transformed with recombinant *Agrobacterium tumefaciens* EHA105 harboring pBEOM-GsSRK-f or pBEOM-GsSRK-t by co-cultivation. The co-cultivated cotyledonary nodes were selected on MS medium containing 0.5 mg L^-1^ glufosinate ammonium for 3–4 weeks. The glufosinate-resistant shoots were transferred onto 1/2MS medium for 2 weeks until roots produced. Then the regenerated seedlings were transferred into soil and grown in a greenhouse under controlled conditions (24–26°C, 70% relative humidity and 16 h light/8 h dark cycles).

Genomic DNA was extracted from the WT and glufosinate-resistant alfalfa plants by using the EasyPure Plant Genomic DNA Kit (TransGen Biotech). PCR analysis was performed by using the specific primers of the *Bar* gene (5′-TGCACCATCGTCAACCACTACATCG-3′ and 5′-CCAGCTGCCAGAAACCCACGTCATG-3′). To determine the expression level of *GsSRK-f* and *GsSRK-t*, total RNA was isolated from the WT and PCR-positive plants by using the RNAprep Pure Plant Kit (TIANGEN), and reversely transcribed into cDNA by using the Goldscript cDNA systhesis kit (Invitrogen). Semi-quantitative RT-PCR was performed by using the *GsSRK* gene specific primers (5′-CAATTATGGTTCTGGCGCG-3′ and 5′-TTGCTTCCTCCGACCAACTC-3′). The *GAPDH* gene in alfalfa (Accession No.: Medtr3g085850) was used as an internal control (5′-GTGGTGCCAAGAAGGTTGTTAT-3′ and 5′-CTGGGAATGATGTTGAAGGAAG-3′).

### Phenotypic Analyses of the *GsSRK* Transgenic Alfalfa under Salt Treatment

Stems were cut from the lignified WT and *GsSRK* transgenic (T_0_ generation) alfalfa, and the stem segments (approximate 1000 stems for each line) were placed in solid dielectric containing vermiculite and perlite (1:1) for approximate 10 days until the new roots appeared. Then the alfalfa plants were transplanted into round pots (8 cm diameter × 10 cm height, 1 plant per pot, 30 pots per tray) containing vermiculite and perlite, irrigated with 3L 1/8 Hoagland solution and grown for 4 weeks in greenhouse at 26°C day/18°C night, and with 16 h light/8 h dark cycles. The WT and transgenic alfalfa seedlings showing similar growth performance were used for salt stress tests. For salt treatment, 3L 1/8 Hoagland solution containing either 0 or 200 or 300 mM NaCl were used for each tray every 2 days for a total of 18 days. After enough soak, excess solution was discarded to prevent waterlogging. Over 30 plants from each line were used for each treatment, and three replicates were carried out for each treatment.

To further verify the effect of *GsSRK-f* and *GsSRK-t* overexpression on alfalfa growth under salt stress, we repeated the stress treatment once again and further checked their growth performance in greenhouse. Briefly, stem segments were cut from the lignified WT and *GsSRK* transgenic alfalfa, and approximate 100 stems were placed in a square pot (40 cm × 25 cm × 12 cm) containing vermiculite and perlite (1:1). The alfalfa plants were irrigated with 1/8 Hoagland solution every 2 days and grown for 6 weeks in greenhouse at 26°C day/18°C night, and with 16 h light/8 h dark cycles. Then 1/8 Hoagland solution containing either 0 or 300 mM NaCl were applied every 2 days for a total of 18 days. After enough soak, excess solution was discarded to prevent waterlogging.

Photos were taken to record the phenotype of the WT and transgenic alfalfa. The shoot length, branch numbers and fresh weight were measured. Shoot length was measured by pulling the plants to a maximum height using centimeter scale. The primary and secondary branches were recorded as the branch numbers. Fresh weight was determined immediately after samples were harvested and blotted with tissue paper to remove the excess water.

To determine the K^+^ and Na^+^ content, the shoots of the WT and transgenic lines were harvested, and dried in a drying oven at 80°C. At least three replicates were carried out with over 1 g dry sample for each line and each experiment. The dried samples were ground thoroughly, and digested in 1 mol L^-1^ HCl overnight. Then, the mixture was centrifuged at 11000 rpm for 3 min and diluted with sterilized milli-Q water. The concentrations of K^+^ and Na^+^ were measured by using an atomic absorption spectrometry instrument (Jia et al., 2017) (Aosong 6400A, Shandong, China).

To monitor the oxidative damages caused by salt stress, the relative ion leakage was measured using a conductivity meter (DDSJ-308A, Shanghai, China) as described (Gibon and Larher, 1997). The MDA (Malon dialdehyde) content was measured by a modified TBA (Thiobarbituric acid) method as described (Peever and Higgins, 1989). The absorbance values at 450, 532, and 600 nm were determined by using an ultraviolet spectrophotometer (UV-2550, Shimadzu, Japan). The SOD (Superoxide Dismutase) activity was determined by monitoring the inhibition of photochemical reduction of NBT (Nitro blue tetrazolium) as described previously (Giannopolitis and Ries, 1977). The reaction mixture contained 1.8 μM riboflavin, 11 mM methionine, 68 μM NBT, 9 μM EDTA, and 22 mM sodium phosphate (pH 7.8) in a final volume of 1.65 mL. One unit of SOD activity was defined as the amount of enzyme required to cause 50% inhibition of the NBT reduction as monitored by an ultraviolet spectrophotometer (UV-2550, Shimadzu, Japan) at 560 nm.

The content of proline was determined with the acidic ninhydrin reagent by measuring the absorbance at 520 nm (Nakazawa et al., 1982). Total chlorophyll content was extracted by using 80% (v/v) acetone, and the absorbance of chlorophyll *a* and *b* were measured at 663 and 645 nm using an ultraviolet spectrophotometer (UV-2550, Shimadzu, Japan) (Arnon, 1949).

For all physiological analysis, samples were harvested from individual plants from different trays under each stress condition. At least 10 individual plants were used for each line and each treatment. All of the above numerical data were subjected to statistical analyses using EXCEL 2010 by Student’s *t*-test.

## Results

### Conserved Domains of the GsSRK Protein

**FIGURE 1:**
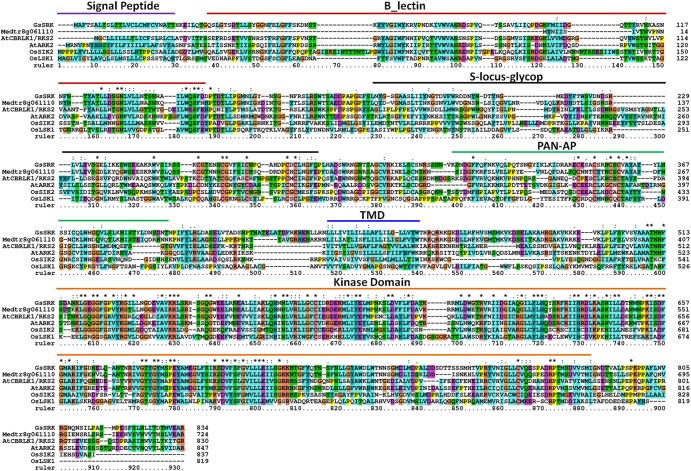
Multiple alignment of GsSRK and its homologous proteins. The full length amino acid sequences of GsSRK and its homologous proteins were used for multiple alignment by using Clustal software. The position of the conserved domains was marked according to GsSRK protein sequence. The accession numbers for GsSRK homologous proteins were Medtr8g061110, LOC_Os07g08860 (OsSIK2), LOC_Os01g47900 (OsLSK1), At1g11350 (AtCBRLK1/RKS2), and At1g65800 (AtARK2).

The receptor-like kinases play crucial regulatory roles in plant growth and stress responses (Bellande et al., 2017; Ye et al., 2017). In a previous study, we elucidated that GsSRK, a receptor like kinase from *Glycine soja*, was a positive regulator of plant tolerance to salt stress (Sun et al., 2013b). As shown in **Figure 1**, GsSRK shows high sequence identity with other characterized RLKs, including AtCBRLK/RKS2 (Kim et al., 2009a,b), AtARK2 (Deb et al., 2014), OsSIK2 (Chen et al., 2013), and OsLSK1 (Zou et al., 2015). In addition, GsSRK displayed very high sequence identity (68%) to its homologous in alfalfa Medtr8g061110. All these proteins consisted of a long extracellular domain at the N-terminus, a transmembrane domain and a C-terminal cytoplasmic kinase domain. The extracellular domain contained a signal peptide, a G-type lectin domain, an S-locus-glycop domain, and a PAN-AP domain (**Figure 1**). GsSRK contained a signal peptide (1–25 aa), a G-type lectin domain (33–149 aa), an S-locus-glycop domain (187–288 aa), a PAN-AP domain (318–394 aa), a transmembrane domain (434–456 aa), and a C-terminal cytoplasmic kinase domain (513–787 aa). These conserved extracellular domains showed that GsSRK belonged to the S-locus LecRLK subfamily, which is characterized by the extracellular G-type lectin and S-locus-glycop domains (Yang et al., 2016).

### Ectopic Expression of *GsSRK* in *Medicago sativa*

**FIGURE 2:**
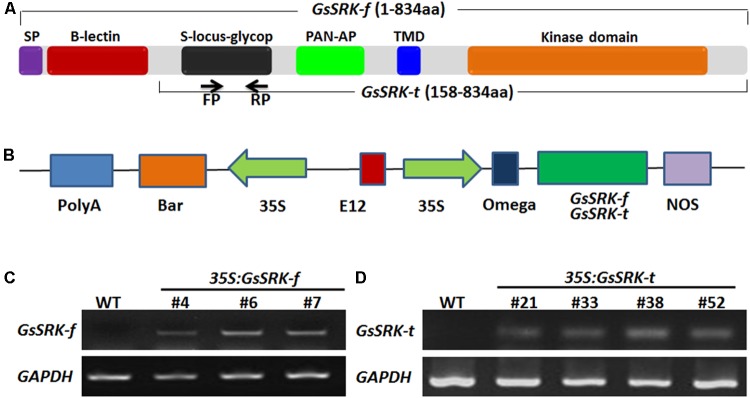
Generation of *GsSRK-f* and *GsSRK-t* transgenic alfalfa. **(A)** Structures showing the conserved domains of *GsSRK-f* and *GsSRK-t*. The arrows labeled with FP and RP indicated the positions of the primers used for semi-quantitative RT-PCR analysis. **(B)** Structures showing the constructs for *GsSRK-f* and *GsSRK-t* overexpression in alfalfa. Construct for *GsSRK-f* overexpression contains the full-length *GsSRK* gene, while construct for *GsSRK-t* overexpression contains a truncated version of *GsSRK-f*, without the N-terminal signal peptide and G-type lectin domain. **(C)** Semi-quantitative RT-PCR identification of *GsSRK-f* transgenic lines. **(D)** Semi-quantitative RT-PCR identification of *GsSRK-t* transgenic lines. The *GAPDH* gene in alfalfa (Accession No.: Medtr3g085850) was used as an internal control.

Several researches have uncovered the key role of the extracellular domain of the S-locus LecRLKs (Naithani et al., 2007; Chen et al., 2013; Zou et al., 2015). The S-locus-glycop domain is linked to self-incompatibility (Zhang et al., 2011). The S-locus-glycop and PAN-AP domains are responsible for dimerization of SRKs (Naithani et al., 2007). However, for now, the function of the G-type lectin domain is still unknown. In this study, we constructed the overexpression vectors carrying the full-length *GsSRK* gene (here termed as *GsSRK-f*, 834 aa) and a truncated version (*GsSRK-t*, 158 aa -834 aa) without the N-terminal signal peptide and G-type lectin domain (**Figures 2A,B**) under the control of the CaMV35S promoter and the E12 enhancer. These two constructs were introduced into alfalfa by *Agrobacterium*-mediated transformation. By using PCR and semi-quantitative RT-PCR analyses, we identified three *GsSRK-f* (#4, #6, and #7) and four *GsSRK-t* (#21, #33, #38 and #52) transgenic lines, respectively (**Figures 2C,D**). The RT-PCR results showed that *GsSRK* was not expressed in the WT plants, but constitutively expressed in all transgenic lines.

### Overexpression of *GsSRK-t* But Not *GsSRK-f* in Alfalfa Results in Morphological Changes

**FIGURE 3:**
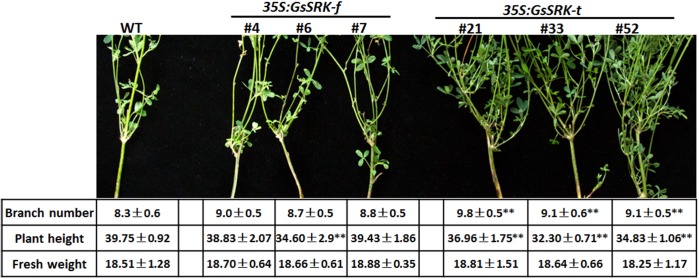
Overexpression of *GsSRK-t* but not *GsSRK-f* in alfalfa results in morphological changes. Pictures show the branching of the WT, *GsSRK-f* and *GsSRK-t* transgenic alfalfa. Detailed information of the branch numbers, plant height, and fresh weight are shown in the table. Data are shown as means ± SE (*n* ≥ 10).

A previous study revealed that overexpression of the extracellular domain of *OsLSK1* altered the panicle architecture and improved grain yield in rice (Zou et al., 2015), indicating the potential role of the extracellular domain of S-locus LecRLKs in regulating plant architecture. Hence, we firstly checked the architecture of the *GsSRK-f* and *GsSRK-t* transgenic alfalfa under normal growth conditions. As shown in **Figure 3**, *GsSRK-t* expression, not *GsSRK-f*, results in morphological changes of transgenic alfalfa plants. In detail, *GsSRK-t* transgenic plants appeared much stronger and had more branches than WT and *GsSRK-f* lines. Quantification analysis further revealed that the plant height of *GsSRK-t* transgenic lines was smaller, but the branch number was much larger than WT and *GsSRK-f* lines (**Figure 3**). As a consequence, *GsSRK-t* transgenic lines displayed similar fresh weight to WT and *GsSRK-f* lines. These morphological changes of transgenic alfalfa suggested an important role of the extracellular G-type lectin domain within *GsSRK* in regulating plant architecture.

### *GsSRK-f* and *GsSRK-t* Overexpression in Alfalfa Contributes to Increased Salt Tolerance

**FIGURE 4:**
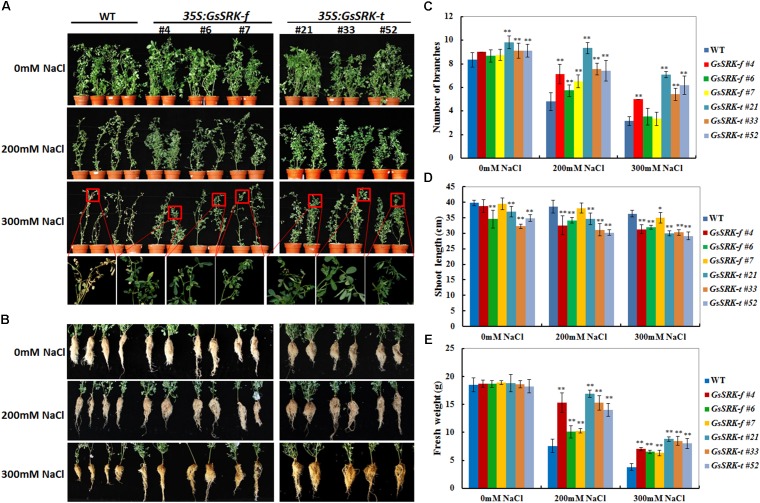
*GsSRK-f* and *GsSRK-t* overexpression in alfalfa contributes to increased salt tolerance. **(A)** Shoot growth performance of the WT and transgenic alfalfa under salt stress. **(B)** Root growth performance of the WT and transgenic alfalfa under salt stress. The WT and transgenic alfalfa seedlings showing similar growth performance were irrigated with 1/8 Hoagland solution containing either 0 or 200 or 300 mM NaCl every 2 days for a total of 18 days. **(C)** Numbers of branches of the WT and transgenic alfalfa under control conditions and salt stress. **(D)** Shoot length of the WT and transgenic alfalfa under control conditions and salt stress. **(E)** Fresh weight of the WT and transgenic alfalfa under control conditions and salt stress. Ten seedlings of each line were used for each experiment. Data are means ± SE (*n* ≥ 10) of three replicates. ^∗^*P* < 0.05; ^∗∗^*P* < 0.01 by Student’s *t*-test.

In order to determine the effect of *GsSRK-f* and *GsSRK-t* overexpression on salt tolerance, the WT and transgenic alfalfa plants were irrigated with 1/8 Hoagland nutrient solution containing either 0 or 200 or 300 mM NaCl for 18 days, respectively. As shown in **Figure 4**, after treated with 200 and 300 mM NaCl, the growth of both WT and transgenic plants was restricted. However, both the *GsSRK-f* and *GsSRK-t* transgenic plants exhibited much better growth performance than WT under salt stress. Salt treatment significantly restricted shoot branching of both WT and *GsSRK-f* plants, but not *GsSRK-t* transgenic lines (**Figure 4A**). Under 300 mM NaCl stress, the leaves of WT plants turned yellow and withered severely, whereas the *GsSRK-f* and *GsSRK-t* transgenic lines displayed green and healthy leaves (**Figure 4A**). Moreover, after salt treatment, roots of the transgenic lines also showed much better growth than that of WT (**Figure 4B**).

To further confirm the better growth of the *GsSRK-f* and *GsSRK-t* transgenic lines, we measured the branching numbers, shoot length, and fresh weight under both control conditions and salt stress. As shown in **Figure 4C**, under control conditions, the *GsSRK-t* transgenic lines displayed more branches than WT and *GsSRK-f* lines. Shoot branching was greatly restricted by salt stress; however, both *GsSRK-f* and *GsSRK-t* transgenic lines displayed more branches than WT. In detail, after 200 mM NaCl treatment for 18 days, the branching numbers of WT plants decreased from 8.3 to 4.8, the average branching numbers of *GsSRK-f* transgenic lines decreased from 8.8 to 6.4. However, the numbers of branches of the *GsSRK-t* transgenic lines only decreased from 9.3 to 8.1. Under 300 mM NaCl stress, the branching numbers of WT and *GsSRK-f* lines were similar, however, *GsSRK-t* transgenic lines displayed more branches. By contrast, salt stress did not severely affect the shoot length (**Figure 4D**). The shoot length of WT plants were 39.75 cm under control conditions, 38.59 cm under 200 mM NaCl and 36.29 cm under 300 mM NaCl treatments. However, the shoot length of the *GsSRK-f* and *GsSRK-t* transgenic lines was smaller than that of WT (**Figure 4D**). As a consequence, after NaCl treatment, both the *GsSRK-f* and *GsSRK-t* transgenic plants displayed higher fresh weight than WT (**Figure 4E**).

To further verify the improved growth of the *GsSRK-f* and *GsSRK-t* transgenic lines, we further checked their growth performance in greenhouse. As shown in **Supplementary Figure S1**, after 300 mM NaCl treatment, the *GsSRK-f* and *GsSRK-t* transgenic plants displayed more branches and much better growth performance than WT (**Supplementary Figure S1**). In conclusion, these morphological changes suggested that overexpression of *GsSRK-f* and *GsSRK-t* increased the salt tolerance of transgenic alfalfa and promoted their growth under salt stress.

### *GsSRK* Overexpression Facilitates to Maintain the Na^+^/K^+^ Balance under Salt Stress

It is well-known that under salt stress, plants have to promote Na^+^ excretion and K^+^ uptake to maintain cytoplasmic Na^+^/K^+^ stable and alleviate the toxicity of excess Na^+^ (Zhu, 2016). In order to evaluate the effect of *GsSRK* overexpression on the Na^+^/K^+^ ion balance, we examined the Na^+^ and K^+^ contents in both the WT and transgenic lines.

**FIGURE 5:**
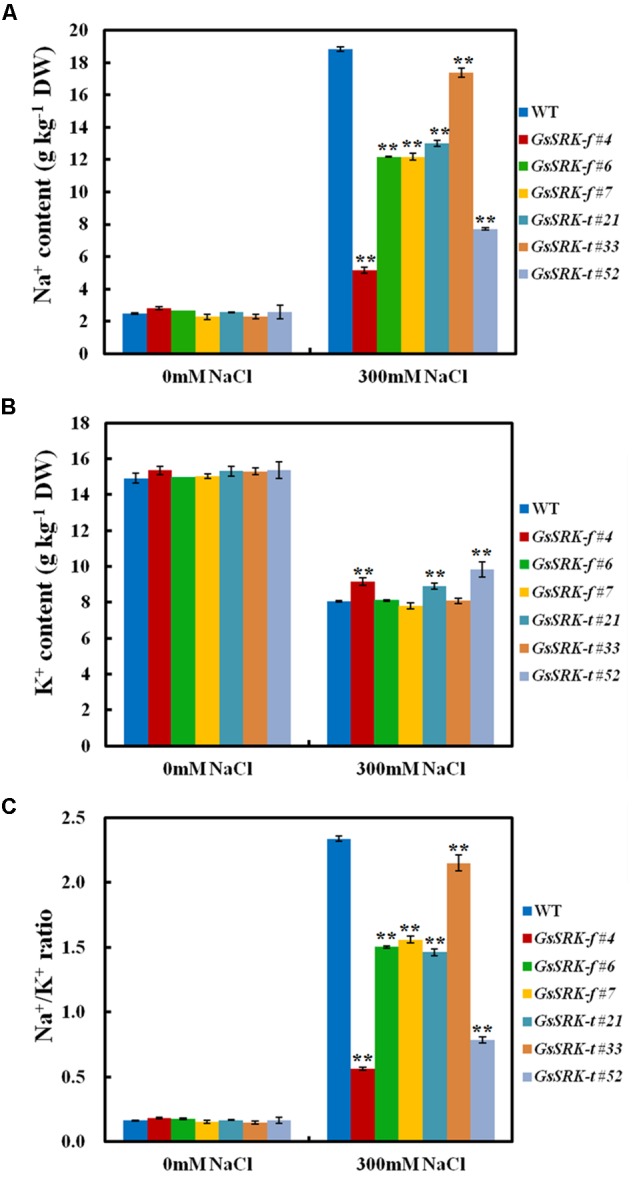
*GsSRK* overexpression facilitates to maintain the Na^+^/K^+^ balance under salt stress. **(A)** The Na^+^ contents of the WT and transgenic alfalfa under control conditions and salt stress. **(B)** The K^+^ contents of the WT and transgenic alfalfa under control conditions and salt stress. **(C)** The Na^+^/K^+^ ratio of the WT and transgenic alfalfa under control conditions and salt stress. Data are means ± SE (*n* ≥ 3) of three replicates. ^∗∗^*P* < 0.01 by Student’s *t*-test.

Under normal condition, both *GsSRK-f* and *GsSRK-t* transgenic lines exhibited similar Na^+^ and K^+^ accumulation (**Figures 5A,B**). Salt stress increased the Na^+^ content but decreased the K^+^ content in plants. However, the Na^+^ content in the *GsSRK-f* and *GsSRK-t* transgenic lines was significantly lower than that in WT plants under salt stress (**Figure 5A**). Furthermore, after salt treatment, *GsSRK-f* (#4) and *GsSRK-t* (#21 and #52) transgenic lines displayed slightly greater K^+^ accumulation (**Figure 5B**). As a consequence, the *GsSRK-f* and *GsSRK-t* transgenic lines showed remarkably decreased Na^+^/K^+^ ratio than WT under salt stress. These results indicate that *GsSRK-f* and *GsSRK-t* overexpression could help plants to maintain low Na^+^/K^+^ ratio and alleviate ion poison under salt stress, possibly by reducing Na^+^ absorbtion, and/or by increasing Na^+^ excretion, and/or by promoting K^+^ uptake.

In addition, even though *GsSRK-t* transgenic lines displayed better salt tolerance than *GsSRK-f* and WT plants, *GsSRK-f* and *GsSRK-t* transgenic lines showed no obvious difference in Na^+^ and K^+^ accumulation, as well as the cytoplasmic Na^+^/K^+^ ratio under salt stress. This finding implies that the deletion of the G-type lectin domain of *GsSRK* does not alter its effect on ion balance under salt stress.

### *GsSRK* Is Involved in ROS Scavenging and Osmotic Regulation under Salt Stress

**FIGURE 6:**
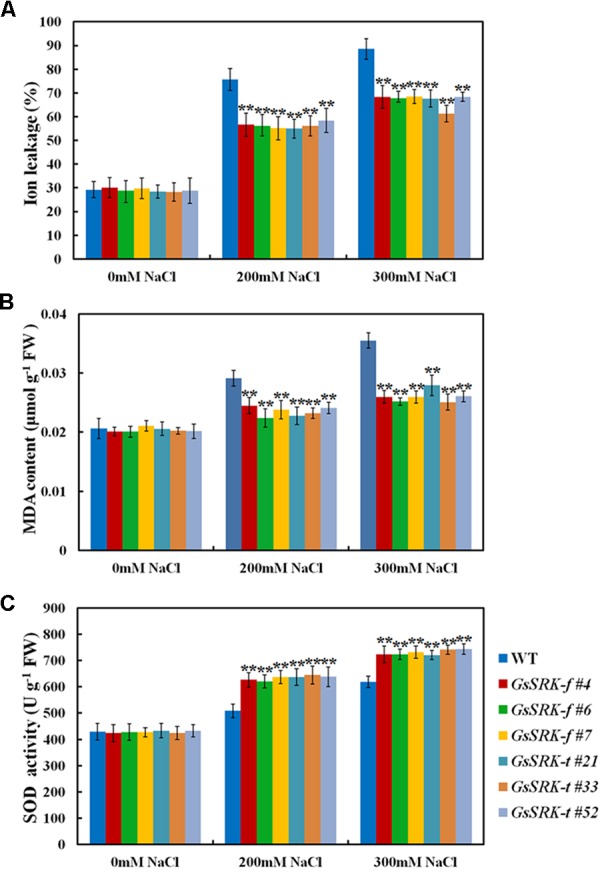
*GsSRK* is involved in regulating ROS scavenging under salt stress. **(A)** The relative ion leakage of the WT and transgenic alfalfa under control conditions and salt stress. **(B)** The MDA content of the WT and transgenic alfalfa under control conditions and salt stress. **(C)** The SOD activity of the WT and transgenic alfalfa under control conditions and salt stress. Data are means ± SE (*n* ≥ 6). ^∗∗^*P* < 0.01 by Student’s *t*-test.

Salt stress leads to reactive oxygen species (ROS) production, which imposes oxidative damages on plant cells (Wang et al., 2015b; Zhang et al., 2016b). To investigate the potential role of *GsSRK* in ROS regulation, we further examined the relative ion leakage and MDA content in WT and transgenic lines to assess the oxidative damages after salt treatment. As shown in **Figure 6**, salt stress significantly increases the relative ion leakage and MDA accumulation. However, both the *GsSRK-f* and *GsSRK-t* transgenic lines showed reduced ion leakage (**Figure 6A**) and MDA content (**Figure 6B**) than WT, indicating that *GsSRK-f* and *GsSRK-t* transgenic lines suffered less ROS damages than WT plants. Besides, we also measured the SOD activity in both WT and transgenic lines. Our results showed that after salt treatment, the SOD activity was increased; however, both the *GsSRK-f* and *GsSRK-t* transgenic lines displayed significantly higher SOD activity than WT (**Figure 6C**). The increased SOD activity in transgenic lines implies that *GsSRK* overexpression could enhance the ROS scavenging and alleviate the ROS damages under salt stress. Notably, no significant difference in the relative ion leakage, MDA accumulation, and SOD activity was observed between the *GsSRK-f* and *GsSRK-t* transgenic lines.

**FIGURE 7:**
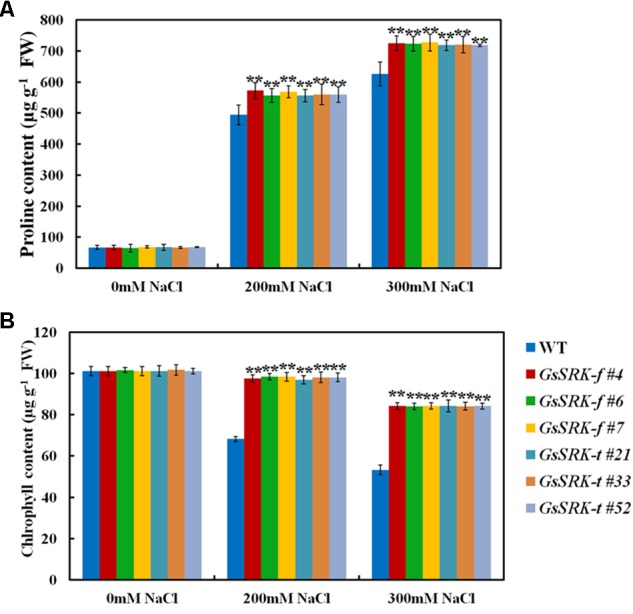
Effect of *GsSRK-f* and *GsSRK-t* overexpression on proline and chlorophyll accumulation. **(A)** The proline content of the WT and transgenic alfalfa under control conditions and salt stress. **(B)** The total chlorophyll content of the WT and transgenic alfalfa under control conditions and salt stress. Data are means ± SE (*n* ≥ 6). ^∗∗^*P* < 0.01 by Student’s *t*-test.

Under salt stress, excess Na^+^ in cytoplasm causes osmotic stress; hence osmotic adjustment is an effective way to deal with salt stress (Wang et al., 2015a; Gharsallah et al., 2016). Proline is one of the important osmotic components, and plays a critical role in protecting plants from salt stress (Bojorquez-Quintal et al., 2014). In this study, we also compared the proline content between WT and transgenic lines. As shown in **Figure 7A**, the proline accumulation is greatly increased under salt stress. However, after salt treatment, the proline content was higher in both the *GsSRK-f* and *GsSRK-t* transgenic lines than WT (**Figure 7A**). In addition, no obvious difference in proline accumulation was found between the *GsSRK-f* and *GsSRK-t* lines.

Moreover, we also determined the chlorophyll content of WT and transgenic lines, because we found that under salt stress, the WT leaves turned yellow and withered, while the *GsSRK-f* and *GsSRK-t* transgenic lines displayed green and healthy leaves (**Figure 4A**). As shown in **Figure 7B**, under salt stress, the chlorophyll content in WT plants decreases greatly, however, the decrease in transgenic lines is much less. The chlorophyll content in transgenic lines was much higher than that in WT under salt stress, which is consistent with the growth performance of WT and transgenic plants (**Figure 7B**). No difference in the chlorophyll content was found between the *GsSRK-f* and *GsSRK-t* transgenic lines.

Taken together, all these results suggested that *GsSRK* overexpression could increase plant salt stress tolerance and promote plant growth under salt stress. *GsSRK-t* transgenic lines displayed much better growth performance than *GsSRK-f* lines.

## Discussion

Even though current researches have suggested the fundamental role of RLKs in stress responses (Marshall et al., 2012; Ye et al., 2017), little information is given as for the S-locus LecRLKs subfamily genes. In a previous research, we isolated an S-locus LecRLK gene from *Glycine soja GsSRK* (Sun et al., 2013b). Similar to other typical S-locus LecRLKs, GsSRK protein contains an intracellular kinase catalytic domain, a transmembrane domain and an extracellular domain consisting of a signal peptide, a G-type lectin domain, an S-locus-glycop domain, and a PAN-AP domain (**Figure 1**).

It is previously reported that the S-locus-glycop domain is linked to self-incompatibility (Zhang et al., 2011), and the S-locus-glycop and PAN-AP domains are responsible for dimerization of the S-locus LecRLKs (Naithani et al., 2007). However, the biological function of the G-type lectin domain is yet to know. Hence, in this study, in order to investigate the role of the G-type lectin domain, we transformed the full-length *GsSRK* and a truncated version deleting the G-type lectin domain into alfalfa (**Figure 2**). By comparing the WT, *GsSRK-f* and *GsSRK-t* transgenic plants, we found that *GsSRK-t* expression resulted in morphological changes of transgenic alfalfa plants, while *GsSRK-f* expression did not (**Figure 3**). *GsSRK-t* transgenic alfalfa had more branches and appeared slightly shorter than WT and *GsSRK-f* lines. These findings indicate a potential involvement of the G-type lectin domain within *GsSRK* in regulating plant architecture.

Previous researches also reported the involvement of the extracellular domain of the S-locus LecRLKs in plant architecture. A previous study revealed that overexpression of the extracellular domain of *OsLSK1* led to larger panicles and taller culm in rice, while the *OsLSK1-f* overexpression and *OsLSK1* RNAi transgenic plants did not show obvious phenotype during the entire rice growth period (Zou et al., 2015). Another study also illustrated the regulation of an S-locus LecRLK gene *OsSIK2* in plant architecture and development (Chen et al., 2013). At the seedling stage, overexpression of the full-length *OsSIK2* (*OsSIK2-f*) and a truncated version (*OsSIK2-t*) deleting the signal peptide, G-type lectin, S-locus-glycop, and partial PAN-AP domain led to a dwarf phenotype, while *OsSIK2* knockout mutant did not display dwarf. Notably, *OsSIK2-t* transgenic plants generated the third leaf earlier than *OsSIK2-f* transgenic lines, and appeared to have a delayed-senescence phenotype. Even though these two studies both uncovered the regulatory role of the S-locus LecRLK extracellular domain in plant architecture and development, it is still unclear which domain within the extracellular region results in these morphological changes. Our results suggest that it is the G-type lectin domain which is linked to the changes of plant architecture.

Previous researches proposed that the phenotype of the *OsLSK1-t* transgenic plants might be caused by GA concentration and signal transduction because Os*LSK1-t* overexpression led to a similar phenotype with the exogenous application of GA (Zou et al., 2015). However, the phenotype (dwarf and more branches) of the *GsSRK-t* transgenic alfalfa is similar to the application of IAA. Furthermore, it is reported that lectins could interact with IAA, and possibly lectin controls the availability of IAA during early seedling stages (Edelman and Wang, 1978; Delatorre et al., 2013). Another research also reported that an S-locus LecRLK in *Arabidopsis AtARK2* was required for auxin-mediated lateral root development under phosphate-starved conditions (Deb et al., 2014). Hence, we proposed that the lectin domain of the S-locus LecRLKs might be responsible for recognition of IAA. It is possible that deletion of the lectin domain of *GsSRK* might block the IAA recognition process. However, further experiments are needed to identify the precise function of the lectin domain in IAA recognition.

*GsSRK* was previously identified as a salt-alkaline stress responsive gene by using the transcript data of *Glycine soja* roots treated with 50 mM NaHCO_3_ (Ge et al., 2010). Our quantitative real-time PCR results confirmed that *GsSRK* expression was induced by environmental stresses, including salt, drought, and ABA (Sun et al., 2013b). In recent years, the expression of the S-locus LecRLK family genes has been reported to respond to various environmental stresses (Vaid et al., 2012; Chen et al., 2013; Shumayla et al., 2016). However, compared with the L-type and C-type LecRLKs, little information is provided concerning the biological function and responsive mechanisms of the S-locus G-type LecRLKs to environmental stress.

Until now, only several researches reported the function of the S-locus G-type LecRLKs in environmental stress responses (Kim et al., 2009b; Chen et al., 2013; Deb et al., 2014). Our previous researches showed that *GsSRK* contributed to salt stress tolerance in transgenic *Arabidopsis* (Sun et al., 2013b). However, the physiological mechanism of *GsSRK* in salt stress responses is still unknown. In this study, we further investigated the physiological function of *GsSRK* in ion homeostasis, ROS scavenging, and osmotic regulation by using transgenic alfalfa. Firstly, under salt stress, the Na^+^ content in *GsSRK* transgenic lines was lower, while the K^+^ content was slightly higher than that in WT (**Figure 5**), indicating that *GsSRK* overexpression helps plants to maintain low Na^+^/K^+^ ratio under salt stress. Secondly, *GsSRK* transgenic lines displayed less ion leakage, lower MDA content, but higher SOD activity than WT (**Figure 6**), implying that *GsSRK* overexpression could alleviate the oxidative damages under salt stress. Thirdly, the proline content was higher in *GsSRK* transgenic lines than WT (**Figure 7**), suggesting that *GsSRK* overexpression affect proline accumulation under salt stress.

Notably, both *GsSRK-f* and *GsSRK-t* were found to be involved in ion homeostasis, ROS scavenging, and osmotic regulation under salt stress. Even though *GsSRK-t* transgenic alfalfa showed more branches and higher fresh weight than *GsSRK-f* lines under salt stress (**Figure 4** and **Supplementary Figure S1**), no obvious difference was observed in the physiological indices between the *GsSRK-f* and *GsSRK-t* transgenic lines (**Figures 5**–**7**). These results suggest that deletion of the G-type lectin domain does not alter the effect of *GsSRK* in ion homeostasis, ROS scavenging, and osmotic regulation under salt stress. The better growth of *GsSRK-t* transgenic plants than *GsSRK-f* lines might be a consequence of the regulation of the lectin domain on plant architecture, or IAA signaling under salt stress. Previous results found that *OsSIK2-t*, a truncated version without the signal peptide, G-type lectin, S-locus-glycop, and partial PAN-AP domain, conferred higher salt tolerance than *OsSIK2-f*, likely through activating different sets of downstream genes (Chen et al., 2013). It is possible that deletion of the GsSRK lectin domain might affect the interaction with IAA, and alter the IAA signaling responsive genes under salt stress. Further researches are needed to precisely characterize the role of the extracellular domain of the S-locus LecRLKs in stress responses.

## Author Contributions

XS, XQ, and YZ designed the research; MS, CC, XQ, SC, BJ, and XS performed the experiments, analyzed the data, and interpreted the results; MS, CC, XQ, BJ, and XS drafted the work; MS, BJ, XS, and YZ edited and revised the manuscript critically. All authors agreed to be accountable for all aspects of the work in ensuring that questions related to the accuracy or integrity of any part of the work are appropriately investigated and resolved and approved the final version to be published.

## Conflict of Interest Statement

The authors declare that the research was conducted in the absence of any commercial or financial relationships that could be construed as a potential conflict of interest.

## Supplementary Material

FIGURE S1 Salt stress tests of *GsSRK-f* and *GsSRK-t* transgenic alfalfa in greenhouse. **(A)** The vertical view to show the growth performance of WT, *GsSRK-f* and *GsSRK-t* transgenic alfalfa under salt stress. **(B)** The side view to show the plant height and branching of WT, *GsSRK-f* and *GsSRK-t* transgenic alfalfa under salt stress. **(C)** Detailed photos to show the growth performance of individual alfalfa plants under salt stress. The WT and transgenic alfalfa seedlings grown in greenhouse were irrigated with 1/8 Hoagland solution containing 300 mM NaCl every 2 days for a total of 18 days. Photographs were taken on the 18^th^ day to show the growth performance of each line.
