# Supplementary figures and images for: Ectopic Expression of GsSRK in Medicago sativa Reveals Its Involvement in Plant Architecture and Salt Stress Responses
Source: Front Plant Sci. 2018 Feb 22;9:226. doi: 10.3389/fpls.2018.00226 (PMC5827113; doi:10.3389/fpls.2018.00226)

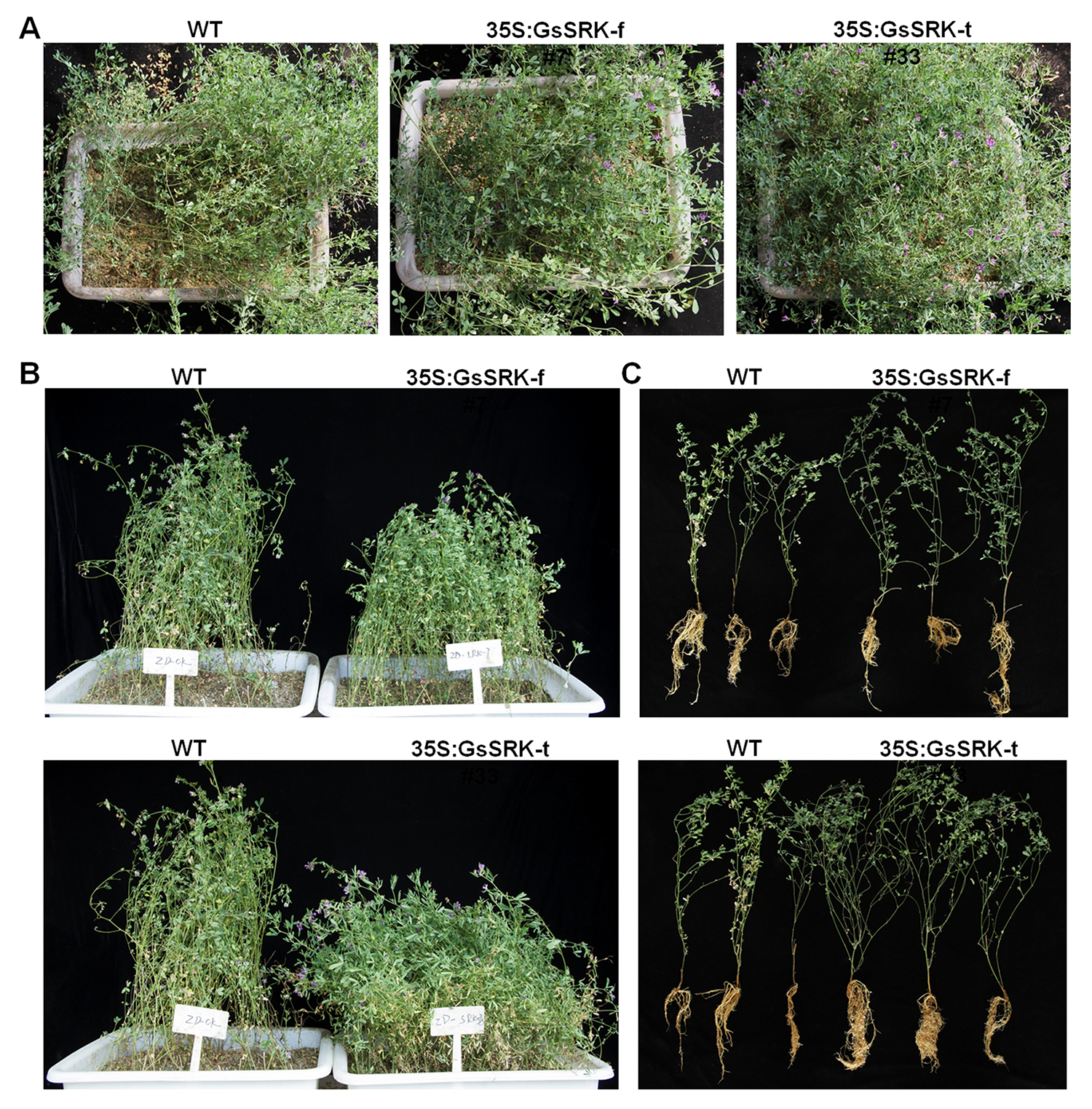

Supplement: FIGURE S1 — Salt stress tests of GsSRK-f and GsSRK-t transgenic alfalfa in greenhouse. (A) The vertical view to show the growth performance of WT, GsSRK-f and GsSRK-t transgenic alfalfa under salt stress. (B) The side view to show the plant height and branching of WT, GsSRK-f and GsSRK-t transgenic alfalfa under salt stress. (C) Detailed photos to show the growth performance of individual alfalfa plants under salt stress. The WT and transgenic alfalfa seedlings grown in greenhouse were irrigated with 1/8 Hoagland solution containing 300 mM NaCl every 2 days for a total of 18 days. Photographs were taken on the 18th day to show the growth performance of each line. [file Image_1.TIF]
